# A Unique Case of COVID-19 Presenting as Focal Seizures With Impaired Awareness

**DOI:** 10.1177/23247096231208771

**Published:** 2023-11-01

**Authors:** Rupam Sharma, Samantha Ratnayake, Hobart Lai, Shikha Mishra, Arash Heidari

**Affiliations:** 1 Kern Medical Center, Bakersfield, CA, USA; 2 David Geffen School of Medicine at UCLA, Los Angeles, CA, USA

**Keywords:** COVID-19 complications, electroencephalography, SARS-CoV-2, seizures/etiology

## Abstract

Severe acute respiratory syndrome coronavirus 2 (SARS-CoV-2) has very rapidly become a global pandemic with millions of confirmed cases worldwide. In early 2021, viral encephalitis was the first neurological complication associated with COVID-19 and since then rise in cases has been reported with this association. A review highlighting 3 potential mechanisms linking a correlation between seizures and COVID-19 was previously reported. Herein described is a unique case of SARS-CoV2 infection that presented with focal seizure with impaired awareness.

## Introduction

Coronavirus disease 2019 (COVID-19) is an infectious disease caused by severe acute respiratory syndrome (SARS)-associated coronavirus 2 (SARS-CoV-2) with millions of confirmed cases worldwide.^1^ Although primary manifestation of COVID-19 infection is pneumonia, several studies have reported the spread to various organs including the central and peripheral nervous system. It has been reported that this virus has neurotropic potential with neurological manifestations noted in several affected individuals.^2^ Encephalitis and seizure associated with COVID-19 have been seen and reported.^3^ The nervous system involvement may be due to the direct action of the virus on the nervous tissue or due to indirect action through the activation of immune-mediated mechanisms.^4^ Viral infections are known to damage the structure and function of the nervous system, presenting as encephalitis, toxic encephalopathy, or postinfectious demyelinating disease.^2^ Herein described is a unique case of SARS-CoV2 infection that presented with focal seizure with impaired awareness.

## Methods

Approval was obtained from the institutional review board of Kern Medical Center. A retrospective review of the patient’s records was performed. A literature search was conducted on PubMed and Google Scholar. The following search terms were applied: COVID-19 and seizures, neurological complications in SARS-CoV-2, encephalitis, and seizure associated with COVID-19.

## Case Presentation

**Image 1.**
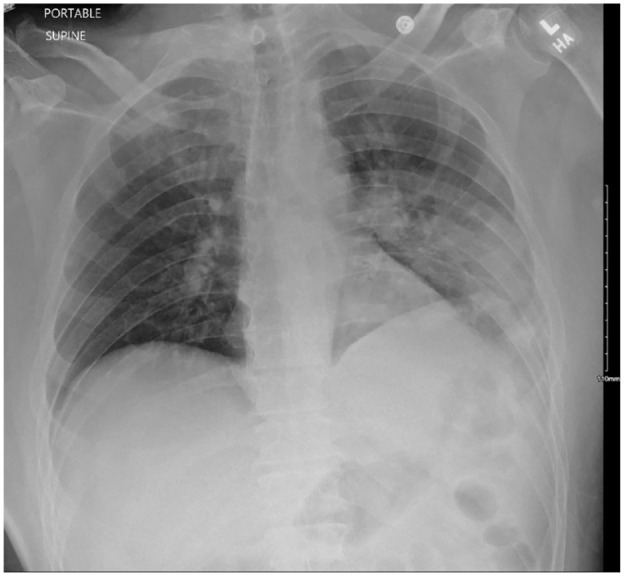
MRI brain: 2 lacunar infarcts seen in right frontal lobe.

**Image 2.**
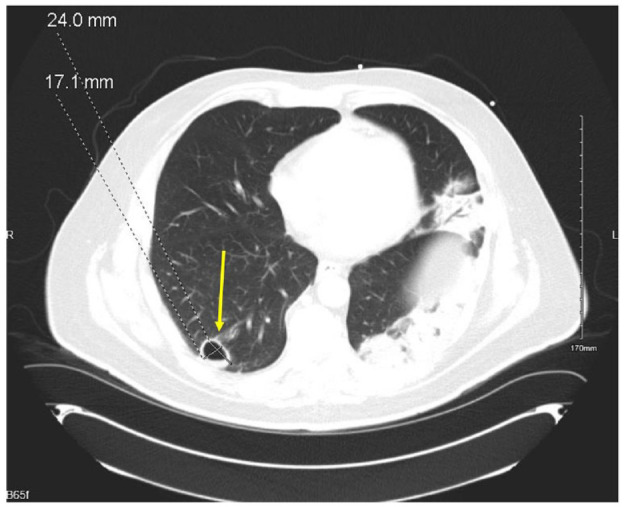
Chest x-ray revealing multifocal pneumonia.

**Image 3.**
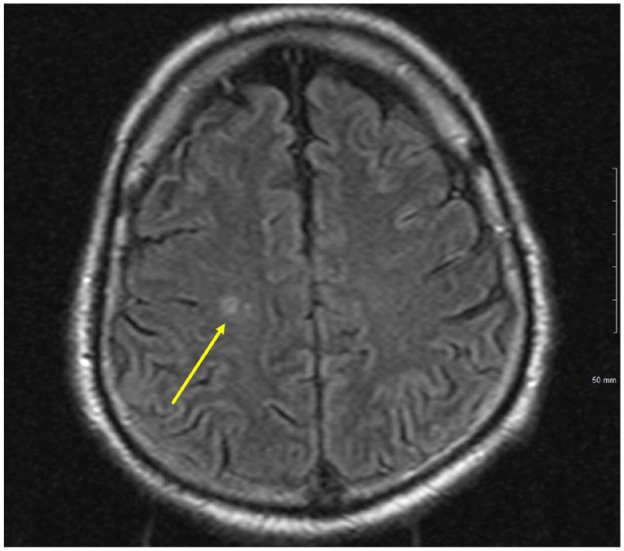
CT chest: right cavitary lesion 2.4 cm × 1.7 cm.

A 54-year-old Hispanic male with no known medical history presented to the emergency department with altered mental status of 1-day duration. Per the patient’s family, he had been complaining of a frontal headache and blurry vision for 3 days prior to the presentation. The day prior to presentation, patient’s son noticed that he was not able to speak appropriately, had difficulty finding his words, and had difficulty with comprehension. His symptoms worsened the following day prompting his sister to bring him to the hospital. Minutes after arrival, he was noted to be oriented only to self and age, with difficulty finding words, and answering all questions with a “yes.” National institutes of health stroke scale was 4: level of consciousness (LOC) command of 1, LOC question of 1, and language/aphasia of 2 due to confusion and inability to name any objects. Initial vitals were significant for blood pressure 160/92, heart rate 108, respiratory rate 17, and afebrile. Laboratory results were significant for glucose of 353 mg/dL and HbA_1c_ of 11.4%. Computer tomography (CT) of brain without contrast showed no evidence of infarction, bleeding, or intracranial lesion. Patient had a witnessed tonic-clonic seizure shortly after admission and was loaded with levetiracetam 1500 mg intravenous one time, followed by 1000 mg twice daily, and 24-hour electroencephalogram (EEG) was ordered. Magnetic resonance imaging (MRI) of brain with and without contrast revealed 2 small areas of slightly increased signal in the subcortical white matter of the superior right frontal lobe that was consistent with subacute lacunar infarctions (Image 1). The CT angiogram of the head and neck showed moderate atherosclerotic plaques and severe intimal thickening of the left carotid bulb and proximal left internal carotid artery without causing significant stenosis. Continuous EEG showed frequent focal seizures in the left occipital cortex, with semiology of eyes deviating to the right, and transient confusion. On assessing for visual fields on hospital day 2, the patient became confused and unable to follow directions. The confusion lasted roughly 4 minutes before he returned to baseline, and he was diagnosed with focal seizure with impaired consciousness. He was therefore loaded with phenytoin 1000 mg, with 100 mg every 8 hours for maintenance, and levetiracetam was increased to 1500 mg twice daily. Despite this regimen, he suffered from a total of 9 additional seizures of focal origin, so he was further loaded with valproic acid 1500 mg once, followed by 750 mg twice daily, with good response. The MRI findings were deemed to be due to uncontrolled diabetes and hypertension and unrelated to his symptoms and seizure due to size and contralateral location. The patient was found to be positive for COVID-19 and was placed on isolation per COVID-19 protocol. He did not require supplemental oxygen and remained on room air throughout admission. His chest x-ray showed multifocal pneumonia (Image 2) and an incidental cavitary lesion in the right lower lobe. These findings were subsequently confirmed with CT of chest showing a right lower lobe cavitary lesion measuring 2.4 cm × 1.7 cm along with confluent sublobar left lower lobe and segmental posterior left upper lobe airspace disease along with additional scattered subsegmental areas of right and left lung airspace disease (Image 3). Pulmonary tuberculosis was ruled out by negative QuantiFERON gold and 3 sputum samples for acid-fast bacilli smears and cultures. Due to his residence in the endemic area for coccidioidomycosis, serology was ordered which resulted as serum immunodiffusion immunoglobulin M (IgM) not detected, immunodiffusion immunoglobulin (IgG) very weakly reactive with a complement fixation titer of <1:2 titer. Given patient resides in a highly endemic area for coccidioidomycosis, diagnosis of pulmonary cavitary coccidioidomycosis was made and patient was initiated on 600 mg fluconazole. This form of pulmonary disease is considered chronic and usually, the IgM immunodiffusion is negative and complement fixation is either negative or low.

Due to the patient’s continued confusion, without a clear culprit lesion found on neuroimaging, in addition to his diagnosis of coccidioidomycosis, a lumbar puncture was performed, with opening pressure 170 mm of H_2_O with unremarkable cerebrospinal fluid (CSF) of 3 white blood cells, 7 red blood cells, glucose of 137 mg/dL, and protein 56 mg/dL. The CSF studies for fungal cultures, coccidioidomycosis serology, adenosine deaminase, west nile IgM and IgG, human simplex virus 1 and 2 DNA PCR, and angiotensin-converting enzyme level were all negative. He was diagnosed with focal seizures with impaired awareness due to COVID-19 infection.

Subsequently, his confusion started to resolve and his seizures were controlled with anti-epileptics, including valproic acid, phenytoin, and levetiracetam. He became fully oriented and was discharged to an acute rehabilitation facility for further physical, occupational, speech, and language therapy. At his 4-week follow-up with a neurologist, he was alert and oriented x3, with recent and remote memory intact, and language was normal.

## Discussion

The etiology of the focal seizures with impaired awareness following the patient’s COVID-19 infection remains unclear. While the patient’s lacunar infarcts may have resulted from complications of COVID-19, poststroke epilepsy is an unlikely explanation due to the contralateral findings on the EEG and MRI.

One possible mechanism for the patient’s seizures is viral encephalitis, a well-known complication of viral infections that can cause seizures.^4-6^ Several recent studies have reported neurological complications associated with SARS-CoV-2 infection, including encephalitis, acute cerebrovascular accidents, ataxia, decreased LOC, seizures, and Guillain-Barre syndrome.^2,7-10^ Mao et al^7^ found that 36.4% of SARS-CoV-2-infected patients had neurological manifestation.

It is hypothesized that the virus’ functional receptor, angiotensin-converting enzyme 2 (ACE2) may play a role in the neurological complications associated with SARS-CoV-2.^11^ The ACE2 is expressed in both the central nervous system and peripheral nervous system, and its downregulation may lead to inflammation and dysfunction in these systems.^12^ The virus may also trigger an autoimmune response or cause direct neuronal damage through hypoxia, thrombosis, or cytokine storm.^13^

This case highlights the importance of considering COVID-19 infection as a potential cause of seizures and other neurological symptoms. Clinicians should remain vigilant in monitoring and managing these complications in patients with COVID-19.
